# Identification of Periopathogens in Atheromatous Plaques Obtained from Carotid and Coronary Arteries

**DOI:** 10.1155/2021/9986375

**Published:** 2021-06-17

**Authors:** Verica Pavlic, Dejan Peric, Ivana Stosovic Kalezic, Marwa Madi, Subraya G. Bhat, Zlata Brkic, Danijela Staletovic

**Affiliations:** ^1^Department of Periodontology and Oral Medicine, Medical Faculty University of Banja Luka, 78000 Banja Luka, Bosnia and Herzegovina; ^2^Department of Periodontology and Oral Medicine, Institute for Dentistry Banja Luka, 78000 Banja Luka, Bosnia and Herzegovina; ^3^Clinic for Dentistry, Faculty of Medicine, University of Priština Kosovska Mitrovica, Kosovska Mitrovica, Serbia; ^4^Department of Preventive Dental Sciences, College of Dentistry, Imam Abdulrahman Bin Faisal University, Dammam, Saudi Arabia; ^5^Clinic of Dental Medicine, Faculty of Medicine of the Military Medical Academy, University of Defense, Belgrade, Serbia

## Abstract

Increasing attention has been paid to the possible link between periodontal disease and atherosclerosis over the past decade. The aim of this study is to investigate the presence of five periopathogens: *Porphyromonas gingivalis* (*P.g.*), *Aggregatibacter actinomycetemcomitans* (*A.a.*), *Tannerella forsythia* (*T.f.*), *Treponema denticola* (*T.d.*), and *Prevotella intermedia* (*P.i.*) in atheromatous plaques obtained from the carotid and coronary arteries in patients who underwent coronary artery bypass graft surgery and carotid endarterectomy. Group I (carotid arteries) consisted of 30 patients (mean age: 54.5 ± 14.8), and group II (coronary arteries) consisted of 28 patients (mean age: 63 ± 12.1). Clinical periodontal examinations consisted of plaque index, gingival index, sulcus bleeding index, and periodontal probing depth and were performed on the day of vascular surgery. The presence of periopathogens in periodontal pockets and atherosclerotic vessels was detected using polymerase chain reaction. In both subgingival plaque and atherosclerotic plaque of carotid arteries, *P.g.*, *A.a.*, *T.f.*, *T.d.*, and *P.i.* were detected in 26.7%, 6.7%, 66.7%, 10.0%, and 20.0%, respectively, while for coronary arteries, *P.g*. was detected in 39.3%, *A.a*. in 25%, *T.f*. in 46.4%, *T.d.* in 7.1%, and *P.i.* in 35.7%. The presence of five periopathogens in carotid and coronary atherosclerotic vessels showed correlation in regard to the degree of periodontal inflammation. The present study suggests the relationship between periodontal pathogenic bacteria and atherogenesis. Further studies are necessary in relation to the prevention or treatment of periodontal disease that would result in reduced mortality and morbidity associated with atherosclerosis.

## 1. Background

Periodontal disease/PD is a chronic inflammatory disease that occurs in the teeth surrounding tissues in response to the presence of bacterial biofilm accumulation and characterized by complex host biofilm interactions [1, 2]. It affects up to 90% of the worldwide population (approximately 75% of the general population is affected by mild forms of periodontal disease including gingivitis, while the remaining 15% of the population has a moderate or severe form of periodontal disease). Therefore, it is ranked as a sixth most prevalent disease affecting humans [1, 2]. The presence of specific, predominantly Gram-negative anaerobic pathogenic periodontal microorganisms/periopathogens, such as *Porphyromonas gingivalis* (*P.g.*), *Aggregatibacter actinomycetemcomitans* (*A.a.*), *Tannerella forsythia* (*T.f.*), *Treponema denticola* (*T.d.*), and *Prevotella intermedia* (*P.i.*), and abnormal host response to periodontal disease are the key determinants of the onset and progression of periodontal disease [3]. In recent years, special attention has been paid to the possibility that the presence of periopathogens may influence systemic health [4–6].

Atherosclerosis is a chronic progressive narrowing of arteries that may lead to occlusion as a consequence of lipid deposition [2]. It underlies coronary heart disease (80%), as well as myocardial and cerebral infarctions, therefore having a big socioeconomic importance [4]. The significant evidence proving the role of chronic inflammation in the pathogenesis of atherosclerosis and the destabilization of existing atheromatous plaques in the arteries has led many researchers to focus their attention to a search for the cause of the inflammation [7]. The link between periodontal disease and atherosclerosis was firstly given in 1963, when 25% higher risk of atherosclerotic plaque formation in a group of patients with periodontal disease was demonstrated [8, 9]. Since then, there is a growing amount of evidence regarding the contribution of chronic inflammation and presence of periopathogens seen in periodontal disease and the enhanced risk of atherosclerosis [7–10]. Increasing evidence over the past decade suggests that periopathogens from periodontal pockets can enter the systemic circulation directly and may be present in peripheral organs, such as atheromatous plaques of different blood vessels. The second mechanism proposed includes increasing levels of inflammatory mediators, such as lipopolysaccharides and other products from periopathogens' cell breakdown that may stimulate inflammatory cytokines, upregulate endothelial adhesion molecules, and induce a prothrombotic environment, enhancing the risk of an atherosclerosis [11]. The causal relationship between periodontal disease and atherosclerosis can be identified through the presence of periopathogens within atheromatous plaques [12].

The aim of the present study was to determine the association between the presences of periopathogens, namely, *P.g.*, *A.a.*, *T.f.*, *T.d.*, and *P.i.*, in subgingival and atheromatous plaques of coronary and carotid arteries in patients with chronic periodontitis, who were hospitalized and underwent surgery, by sampling DNA extract and amplification by polymerase chain reaction (PCR).

## 2. Мaterials and Methods

### 2.1. Patients

A total of 58 patients (male: 42, female: 16) with chronic periodontal disease and atherosclerosis participated in this study. Patients were divided into two groups depending on the atherosclerotic blood vessel, either carotid or coronary arteries. Group I consisted of 30 patients (male: 22, female: 8) from 32 to 83 years of age (mean age: 54.5 ± 14.8 years) scheduled for carotid endarterectomy. Group II consisted of 28 patients (male: 20, female: 8) from 28 to 94 years of age (mean age: 63 ± 12.1 years) with coronary artery disease scheduled for coronary artery bypass graft surgery (CABG).

Regarding periodontal disease, patients were recruited only if they were with at least 4 periodontal pockets. Periodontal disease (PD) was diagnosed if the subject exhibited clinical attachment level (CAL) > 1 mm and periodontal pocket depth (PPD) > 3 mm, at least at three sites in two different quadrants. According to CAL, patients with diagnosed PD were classified into two subgroups: patients with moderate chronic periodontitis (CP) (CAL = 3-4 mm) and severe CP (CAL ≥ 5 mm). PD was defined as localized or generalized depending on the number of affected sites [13]. Periodontal examination was performed by one trained and calibrated periodontist (D.S.). Periodontal and surgical interventions were performed in the Clinic of Dental Medicine, Faculty of Medicine, Military Medical Academy, Belgrade; Clinic of Dental Medicine, Faculty of Medicine, Kosovska Mitrovica; and Clinic for Vascular and Endovascular Surgery, Clinical Center Serbia.

The exclusion criteria were smoking, pregnancy, presence of systemic diseases, use of medication (antibiotic or corticosteroids), and periodontal treatment within the past 3 months. The medical and dental history of each subject was obtained by interwiev. Patients fulfilling the inclusion criteria were fully informed about the study and signed an informed consent form that was approved by the Ethics Committee of the Medical Faculty Kosovska Mitrovica, Priština.

### 2.2. Subgingival and Atheromatous Plaque Sample

On the same day of the surgical intervention for carotid endarterectomy and CABG, a complete periodontal examination was performed. Clinical examinations included plaque index (PI) (according to Silness Lӧe), gingival index (GI) (according to Lӧe Silness), sulcus bleeding index (SBI) (according to Mühlemann Son), and periodontal pocket probing depth (PPD) [13–16]. The subgingival plaque samples were collected using the paper point technique (Periopaper, Amityville, Pro Flow, NY, USA) from the bottom of two out of four present periodontal pockets. Each sample site was isolated with cotton rolls, gently scaled supragingivaly and air dried. A sterile paper point was inserted into the apical extent of each selected pocket, kept for 60 seconds, and transferred immediately to a sterile Eppendorf tube and kept on -70°C until the analysis.

The atheromatous plaque samples were obtained during the surgery, wherein the surgeon excised one or two small bits of atherosclerotic plaque from the edge of the blood vessel. In order to eliminate the blood contamination, the plaque samples were placed in a sterile Eppendorf tube with Tris-EDTA as a transport medium, mixed gently, and kept on -20°C until DNA preparation.

### 2.3. PCR Analysis

16S rRNA PCR amplification was carried out to detect the presence of *P.g.*, *A.a.*, *T.f.*, *T.d.*, and *P.i.* in periodontal pockets and atherosclerotic vessels. The positive controls (American Type Culture Collection (ATCC)) consisted of DNA from pure cultures: *P.g.*—ATCC 33277, *A.a.*—ATCC 33384, *T.f.*—ATCC 43037, *T.d.—*ATCC 35405, and *P.i.*—ATCC 33563. PCR primers of microorganisms in the study are as listed in Table 1. Colonies obtained from cultures were suspended in sterile water and centrifuged and subjected to DNA extraction (positive control). Sterilized distilled water served as the negative control. 25 *μ*l of aqueous mixture containing 2.5 *μ*l of PCR buffer, 2.5 mM MgCl_2_, 0.2 mM dNTPs, 0.2 *μ*M of species specific primers, 1 U of DreamTaq DNA polymerase (all products from Thermo Fisher Scientific™; Waltham, MA, USA), and 5 *μ*l of bacterial DNA isolate was used for the reaction. The temperature profile of the bacteria was 95°C (3 min), 35 cycles of 94°C (1 min), 60°C (1 min), and 72°C (1 min) and final extension at 72°C (7 min). The PCR reaction was carried out using a PCR thermocycler (PeqSTAR, PeqLAB Biotechnologie GmbH, Germany). After amplification, 10 *μ*l aliquot of the amplified PCR product was subjected to electrophoresis in 8% polyacrylamide gel (0.5 x TAE buffer), stained with ethidium bromide, and finally visualized and photographed after exposure to UV light.

### 2.4. Statistical Analysis

The association between the periopathogens in the subgingival and atherosclerotic plaque samples was analyzed by calculating agreement statistics (absolute percentage agreement and Cohen's kappa statistic). The difference in average levels of various periodontal parameters between the patients with periopathogens present in both periodontal and arterial samples and those patients with negative results was tested using the Wilcoxon-Mann-Whitney test. *P* values less than 0.05 were considered statistically significant. All analyses were performed within the statistical software environment R (v4.0.2; R Core Team 2018), by using package irr [17].

## 3. Results

A total of 58 patients (male: 42, female: 16) with periodontal disease and atherosclerosis participated in this study. Totally, 58 atherosclerotic plaque samples (30 from carotid and 28 from coronary arteries) and 58 subgingival plaque samples were examined and compared for the presence of five periopathogens (*P.g.*, *A.a.*, *T.f.*, *T.d.*, and *P.i.*).

The presence of DNA of five periopathogens in subgingival and atheromatous plaques of carotid and coronary arteries is presented in Tables 2 and 3.

In all cases, the bacterial species found in atherosclerotic plaques were also found in the subgingival plaques, although the presence of the periopathogens in subgingival plaque was not always associated with its presence in the atheromatous plaques of the same patients.

The frequencies of bacteria in subgingival versus atherosclerotic samples of carotid arteries were as follows: *P.g.* (53.3%: 26.7%), *A.a.* (36.7%: 6.7%), *T.f.* (80%: 66.7%), *T.d.* (33.3%: 10.0%), and *P.i.* (76.7%: 20.0%) (Table 2). We found a significant agreement of *T.f.* in subgingival plaque and carotid plaque samples (Table 2). The frequencies of bacteria in subgingival versus atherosclerotic samples of coronary arteries were as follows: *P.g.* (57.1%: 39.3%), *A.a.* (42.9%: 25%), *T.f.* (82.1%: 46.4%), *T.d.* (10.7%: 7.1%), and *P.i.* (67.9%: 35.7%) (Table 3). We found a significant agreement of *P.g.*, *A.a.*, and *T.d.* in subgingival plaque and coronary plaque samples (Table 3).

The present study further analyzed the mean value of the selected periodontal parameters, namely, plaque index, gingival index, sulcus bleeding index, and periodontal pocket depth in patients positive to the presence of periopathogens in carotid and coronary atheromatous plaques (Tables 4 and 5). As for relationship between the presences of periopathogens in the carotid atheromatous plaques, all clinical periodontal parameters analyzed were nonsignificant (Table 4). However, the results showed a statistically significant relationship between the presences of *T.f.* in the carotid atherosclerotic plaque with periodontal pocket depth values, while all other periodontal parameters analyzed were nonsignificant (Table 5).

## 4. Discussion

PD represents chronic inflammation in tooth-supportive tissues (periodontal ligament, connective tissue, and alveolar bone); that, if left untreated, leads to periodontal pocket formation and consequent bone loss [18]. It has been suggested that periodontitis-associated bacteraemias and systemic dissemination of inflammatory mediators produced in the periodontal tissues may cause a systemic inflammation. To date, many authors have demonstrated such a relationship [19, 20]. Atherosclerosis, as a progressive disease of the medium and large elastic and muscular arteries, can lead to ischemic lesions of the brain, heart, or extremities and can result in thrombosis and infarction of affected vessels [7–10]. Mechanisms that have been proposed to explain the link between PD and atherosclerotic cardiovascular disease include the inflammatory pathways common to both diseases (increased levels of white blood cells, C-reactive protein/CRP, fibrinogen, intercellular adhesion molecule-1, and proinflammatory cytokines). Additionally, both diseases share similar risk factors such as smoking, poor oral hygiene, diabetes mellitus, obesity, stress, and reduced physical activities [21].

Among 58 patients included in this study, 42 of them (72.4%) were males, which make the prevalence of periodontal disease and atherosclerosis higher in man. Patients' mean age was 58.8 years. That is in correlation with common understanding of periodontal disease's progress. The age itself is not a predetermining risk factor for periodontal disease, but due to the lower number of elastic and collagen fibers as well as mitotic activity of fibroblasts, it is usually seen in adults over 40 years.

This study has proved the presence of peripathogen DNA in atheromatous plaques of coronary and carotid atheromatous and subgingival plaque samples of the same patients. The results are suggesting that periopathogens from subgingival plaque are most likely invading the systemic circulation and therefore were detected in atherosclerotic plaques of nearby heart blood vessels, suggesting its impact on the progression of atherosclerosis.

These results are in accordance with many published studies on this topic [20–31]. The data of this study were consistent with those reported by Haraszthy et al. [20] (26% for *P.g.*, 18% for *A.a.*, 30% positive for *T.f*., and 14% for *P.i.*), Nakano et al. [21] by specific PCR (20% for *P.g.*, 35% for *A.a.*, and 20% *T.d.*), Figuero et al. [25] by nested PCR (78.6% for *P.g.*, 66.7% for *A.a.*, and 61.9% *T.f.*), and Ohki et al. [26] (3.4% for *P.g.*, 19.7% for *A.a.*, and 2.3% *T.d.*). In contrast to the present study and the results of other authors cited above, Cairo et al. [12], when examining 40 samples of atherosclerotic plaques (obtained after carotid endarterectomy) by PCR, did not detect the presence of any periodontal pathogenic bacteria. Aimetti et al. [32] did not isolate any periopathogens in samples taken from atherosclerotic carotid arteries of patients with periodontal disease. These discrepancies in the results from different studies may be associated with the study population, host immune response, and varying methods of sampling and laboratory analysis [27].

In our study, the presence of five periopathogens in carotid and coronary atherosclerotic vessels showed correlation in regard to degree of periodontal inflammation (Tables 4 and 5). Even though most of these correlations were not found to be significant, the prevalence of almost all periopathogens was higher in patients with moderate and severe periodontal disease when compared to patients with average PPD. The possible explanation for this correlation could be that moderate to severe periodontitis increases the level of systemic inflammation. Consequently, periodontal treatment could efficiently reduce clinical signs of the disease and decrease the level of systemic inflammatory mediators. Therefore, further studies with a larger number of patients related to prevention or treatment of periodontal disease that would result in reduced mortality and morbidity associated with atherosclerosis are necessary. Further *in vitro*, *in vivo*, and clinical studies with precise bacterial quantification with longer follow-up are essential in order to confirm the causal relationship between PD and atherosclerosis.

## 5. Conclusion

The present study suggests the relationship between periodontal pathogenic bacteria and atherogenesis. Even though the presence of periopathogens may not be the only factor that causes inflammatory disease associated with atherosclerosis, it should be considered a potential risk factor.

## Figures and Tables

**Table 1:** Bacteria primer sequences used in the polymerase chain reaction (PCR) detection.

Periopathogens	Product size
*Porphyromonas gingivalis* (*P.g.*) CAA TAC TCG TAT CGC CCG TTA TTC	400 bp
*Aggregatibacter actinomycetemcomitans* (*A.a.*) CAC TTA AAG GTC CGC CTA CGT GC	600 bp
*Tannerella forsythia* (*T.f.*) GTA GAG CTT ACA CTA TAT CGC AAA CTC CTA	840 bp
*Treponema denticola* (*T.d.*) TAA TAC CGA ATG TGC TCA TTT ACA T TCA AAG AAG CAT TCC	316 bp
*Prevotella intermedia* (*P.i.*) GTT GCG TGC ACT CAA GTC CGC C	660 bp

**Table 2:** Presence of periopathogens in subgingival and atheromatous plaque of carotid arteries.

Periopathogens	Subgingival plaque	Atheromatous plaque		% agreement	Kappa	*P* value
		No	Yes			
Porphyromonas gingivalis (*P.g.*)	No	14	0	73.3	0.48	0.002
	Yes	8	8			

Aggregatibacter actinomycetemcomitans (*A.a.*)	No	19	0	70.0	0.22	0.054
	Yes	9	2			

Tannerella forsythia (*T.f.*)	No	6	0	86.7	0.67	<0.001
	Yes	4	20			

Treponema denticola (*T.d.*)	No	20	0	76.7	0.36	0.010
	Yes	7	3			

Prevotella intermedia (*P.i.*)	No	7	0	43.3	0.14	0.131
	Yes	17	6			

**Table 3:** Presence of periopathogens in subgingival and atheromatous plaque of coronary arteries.

Periopathogens	Subgingival plaque	Atheromatous plaque		% agreement	Kappa	*P* value
		No	Yes			
Porphyromonas gingivalis (*P.g.*)	No	12	0	82.1	0.65	<0.001
	Yes	5	11			

Aggregatibacter actinomycetemcomitans (*A.a.*)	No	16	0	82.1	0.62	<0.001
	Yes	5	7			

Tannerella forsythia (*T.f.*)	No	5	0	64.3	0.32	0.022
	Yes	10	13			

Treponema denticola (*T.d.*)	No	25	0	96.4	0.78	<0.001
	Yes	1	2			

Prevotella intermedia (*P.i.*)	No	9	0	67.9	0.42	0.007
	Yes	9	10			

**Table 4:** Clinical periodontal parameters for carotid arteries.

Variable	*P.g.* (+)	*P.g.* (-)	*P* value	*A.a.* (+)	*A.a.* (-)	*P* value	*T.f.* (+)	*T.f.* (-)	*P* value	*T.d.* (+)	*T.d.* (-)	*P* value	*P.i.* (+)	*P.i.* (-)	*P* value
*Plaque index (PI)*															
Mean	2.6	2.33	0.251	1.8	2.47	0.182	2.58	2.25	0.173	2.6	2.4	0.711	2.62	2.64	1
SD	0.47	0.58		0.42	0.53		0.52	0.46		0.52	0.54		0.44	0.49	
*Gingival index (GI)*															
Mean	2.79	2.39	0.038	2	2.57	0.433	2.59	2.45	0.296	2.9	2.47	0.06	2.73	2.73	0.717
SD	0.18	0.53		1.13	0.39		0.46	0.39		0.1	0.47		0.23	0.39	
*Sulcus bleeding index (SBI)*															
Mean	4.06	3.81	0.607	2.95	4.04	0.15	4.05	3.5	0.076	3.97	3.82	0.891	4.08	4.29	0.667
SD	0.75	0.91		1.34	0.69		0.81	0.56		1.05	0.77		0.71	0.69	
*Periodontal pocket depth (PPD)*															
Mean	5.63	4.86	0.254	3.5	5.26	0.082	5.25	4.83	0.518	6	5	0.23	5.83	5.86	0.431
SD	0.52	1.46		0.71	1.19		1.21	1.33		1	1.26		0.41	1.35	

**Table 5:** Clinical periodontal parameters for coronary arteries.

Variable	*P.g.* (+)	*P.g.* (-)	*P* value	*A.a.* (+)	*A.a.* (-)	*P* value	*T.f.* (+)	*T.f.* (-)	*P* value	*T.d.* (+)	*T.d.* (-)	*P* value	*P.i.* (+)	*P.i.* (-)	*P* value
*Plaque index (PI)*															
Mean	2.53	2.11	0.057	2.73	2.15	0.058	2.58	2.22	0.194	2.5	2.26	0.53	2.47	2.44	1.000
SD	0.65	0.59		0.23	0.68		0.44	0.6		0.71	0.6		0.43	0.53	
*Gingival index (GI)*															
Mean	2.58	2.36	0.172	2.32	2.28	0.085	2.37	2.38	0.103	2.85	2.45	0.394	1.65	2.49	0.675
SD	0.59	0.85		0.62	0.76		0.27	0.79		0.21	0.72		0.31	0.79	
*Sulcus bleeding index (SBI)*															
Mean	3.93	3.29	0.117	3.65	3.38	0.252	4.09	3.07	0.086	4.45	3.48	0.128	3.85	3.77	0.82
SD	0.88	0.97		1.23	1.02		0.6	1.2		0.64	0.86		0.67	0.8	
*Periodontal pocket depth (PPD)*															
Mean	5.73	4.38	0.015	5.25	4.59	0.021	6.08	4.33	0.004	6.5	4.77	0.21	6	4.8	0.057
SD	1.35	1.39		2.06	1.28		1.12	0.82		2.12	1.24		1.25	1.23	

## Data Availability

Data are available from the corresponding author upon request.
